# LncRNA-mediated cartilage homeostasis in osteoarthritis: a narrative review

**DOI:** 10.3389/fmed.2024.1326843

**Published:** 2024-02-21

**Authors:** Li Zhang, Hejin Zhang, Qian Xie, Haiqi Feng, Haoying Li, Zelin Li, Kangping Yang, Jiatong Ding, Guicheng Gao

**Affiliations:** ^1^Department of Orthopedics, the Second Affiliated Hospital, Jiangxi Medical College, Nanchang University, Nanchang, China; ^2^The First Clinical Medicine School, Nanchang University, Nanchang, China; ^3^The Second Clinical Medicine School, Nanchang University, Nanchang, China; ^4^The Third Clinical Medicine School, Nanchang University, Nanchang, China; ^5^Queen Mary School, Nanchang University, Nanchang, China

**Keywords:** lncRNA, cartilage, cartilage homeostasis, osteoarthritis, targeted therapy

## Abstract

Osteoarthritis (OA) is a degenerative disease of cartilage that affects the quality of life and has increased in morbidity and mortality in recent years. Cartilage homeostasis and dysregulation are thought to be important mechanisms involved in the development of OA. Many studies suggest that lncRNAs are involved in cartilage homeostasis in OA and that lncRNAs can be used to diagnose or treat OA. Among the existing therapeutic regimens, lncRNAs are involved in drug-and nondrug-mediated therapeutic mechanisms and are expected to improve the mechanism of adverse effects or drug resistance. Moreover, targeted lncRNA therapy may also prevent or treat OA. The purpose of this review is to summarize the links between lncRNAs and cartilage homeostasis in OA. In addition, we review the potential applications of lncRNAs at multiple levels of adjuvant and targeted therapies. This review highlights that targeting lncRNAs may be a novel therapeutic strategy for improving and modulating cartilage homeostasis in OA patients.

## 1 Introduction

Articular cartilage is a highly structured tissue that serves various functions (1). Cartilage homeostasis is an essential endostatic process in tissues with low cell turnover (e.g., cartilage), and with age, cartilage undergoes degeneration and denaturation, a process known as cartilage aging and overuse, which leads to a loss of cartilage homeostasis. Cartilage imbalance can lead to the development of osteoarthritic (OA) lesions. OA is a whole-joint disease involving all joint tissues: subchondral bone remodeling (2), meniscal degeneration (3), inflammation and fibrosis of both the infrapatellar fat pad (4) and synovial membrane (5). Aging or overuse of cartilage results in the loss of cartilage homeostasis. Cartilage homeostasis refers to the internal regulatory mechanism that maintains the stability of chondrocytes.

The incidence of OA increases progressively with age, with the incidence of knee OA approaching 1% per year in women aged 70–89 years (6). OA is not only the result of age-related wear and tear of the cartilage but also the pathophysiology of OA involving the entire joint structure. OA affects almost all joint tissues, and the main symptoms are degeneration and loss of articular cartilage (7, 8). As the disease progresses, further symptoms include synovitis, meniscal tears, subchondral bone sclerosis and osteophytes. The onset of OA progressively worsens with age, and the patient’s healthy life expectancy decreases. In terms of biomechanics, the mechanical properties of cartilage tissue are affected by OA, e.g., the modulus of elasticity and coefficient of friction may be altered. Biochemically, the synthesis and degradation of proteins, glycoproteins and other biomolecules in cartilage tissue may be disrupted, leading to degeneration and inflammation (9). There is no cure for OA, but by identifying the factors that affect cartilage homeostasis and the pathways involved in homeostasis, we can find appropriate treatments to avoid OA (10–12).

Recent research has shown that long noncoding *RNAs* (lncRNAs) can play a role in a number of physiological and pathological processes, including certain disorders, such as osteoarthritis (OA) (13). LncRNAs not only affect transcriptional regulation, chromatin remodeling, and the regulation of gene expression in cells but also regulate cell proliferation, differentiation, the inflammatory response, extracellular matrix metabolism, and autophagy in OA (14). Several lncRNAs associated with the inflammatory response, such as *MALAT1* (15), *H19* (16) and *NEAT1* (17), can be involved in the expression of inflammatory factors and regulation of the inflammatory response by interacting with transcription factors or RNA regulatory factors. Several lncRNAs, such as ANCR, lncRNA-HOTAIR and lncRNA-XIST, are also potentially involved in the metabolism and repair of bone and joint tissues (18–20). They can regulate cell proliferation and differentiation and promote the synthesis of bone matrix and the degradation of extracellular matrix, thereby participating in the metabolism and repair of bone and joint tissues. The use of these characteristics may provide new ideas for the treatment of OA.

LncRNAs may play a role in the diagnosis, treatment and prognosis of OA, but the intricate mechanisms of lncRNAs in OA are still unclear (21). Therefore, this paper reviews the regulatory network of lncRNAs in OA and explores the potential value of these RNAs as biomarkers or participants in clinical treatment.

## 2 Search methodology for narrative review

(1) Rationale for a narrative review.

In the field of the molecular mechanisms underlying OA, the role of lncRNAs in maintaining cartilage homeostasis has garnered increasing attention. While systematic reviews and meta-analyses are valuable for synthesizing quantitative data, a narrative review offers a unique opportunity to comprehensively explore the intricate molecular pathways and regulatory networks involving lncRNAs in the context of OA. By synthesizing evidence from diverse sources and providing a coherent narrative, this review aims to offer a deeper understanding of the complex interplay between lncRNAs and cartilage homeostasis in OA.

(2) Clarity of boundaries, scope, and definitions.

This narrative review will focus specifically on the molecular mechanisms through which lncRNAs contribute to the maintenance of cartilage homeostasis in the context of OA. The boundaries of the review will be clearly delineated to encompass relevant studies that elucidate the involvement of lncRNAs in the regulation of key cellular processes within chondrocytes and the extracellular matrix. Definitions of key concepts and terms will be provided to ensure clarity and precision in the discussion of molecular pathways and regulatory mechanisms. To conduct a literature review on the topic of long noncoding RNAs, OA, and cartilage homeostasis, we utilized the Scopus, PubMed, and Web of Science (WOS) databases. The search strategy included a combination of keywords and MeSH terms related to the three topics. The search terms used were “long noncoding RNA,” “lncRNA,” “osteoarthritis,” “cartilage homeostasis,” “chondrocytes,” and “joint disease.” The search was limited to articles published in English, and no date restrictions were applied.

(3) Justification for inclusion and exclusion criteria.

The inclusion criteria will prioritize studies that provide mechanistic insights into the roles of specific lncRNAs in modulating the expression of genes involved in cartilage maintenance and repair. Studies employing diverse experimental approaches, including *in vitro* and *in vivo* models, will be included to capture the breadth of evidence. The exclusion criteria will be applied to studies that lack relevance to the molecular mechanisms of lncRNA-mediated cartilage homeostasis or those with insufficient methodological rigor.

(4) Reflexivity and a saturation statement.

Throughout the review process, reflexivity will be maintained by critically examining the potential biases and limitations inherent in the selected literature. Saturation will be considered in the context of the depth and diversity of evidence, ensuring that the review captures the full spectrum of relevant findings.

(5) Details on the analysis and interpretation.

The analysis involved synthesizing the findings from selected studies to elucidate common themes and patterns in the molecular mechanisms through which lncRNAs influence cartilage homeostasis in OA. Emphasis will be placed on the interconnected signaling pathways and regulatory networks involving lncRNAs, with a focus on identifying potential therapeutic targets and strategies. This interpretation will involve critically evaluating the implications of the findings within the broader context of OA pathogenesis and the development of novel therapeutic interventions targeting lncRNAs.

## 3 Cartilage homeostasis and OA

In OA and cartilage homeostasis, ROS (reactive oxygen species) are oxidative stress molecules that can affect chondrocyte function by oxidizing proteins and DNA (22). Transcription factors are a class of proteins that regulate the transcription of genes, thereby affecting the biological function of chondrocytes (23). Growth factors are a class of signaling molecules that can promote chondrocyte proliferation and differentiation (24). ROS, transcription factors and growth factors play important regulatory roles in OA and cartilage homeostasis, and their balanced and coordinated relationship is critical for maintaining cartilage health and homeostasis. The study of these factors contributes to an in-depth understanding of the pathogenesis of OA and provides a theoretical basis for the development of new approaches to treat OA.

### 3.1 Reactive oxygen species

Reactive oxygen species (ROS) are involved in regulating chondrocyte proliferation and differentiation and influencing the maintenance of cartilage homeostasis. Increased mitochondrial-derived ROS are associated with age-related mitochondrial dysfunction and increased chondrocyte ROS. Superoxide dismutases 2 (SOD2) play a key role in defending against ROS (25). Overexpression of SOD2 or targeted mitochondrial reinforcement therapy reduces OA progression, while deficiency of the antioxidant gene transcriptional regulator Nrf2 results in more severe OA. Focusing on novel therapeutic strategies to enhance specific antioxidant systems, such as mitochondrial ROS, may be beneficial for slowing the progression of age-related OA (26). An *in vitro* study of human OA chondrocytes revealed impaired mitochondrial function and elevated ROS levels (27).

Antioxidant therapy may help treat inflammatory structural changes in bones and joints by preventing ROS-induced tissue remodeling and synovial inflammation, although it may not relieve symptoms (28). Further investigation of ROS has shown that various OA elements can stimulate chondrocyte production and that ROS act as secondary messengers to regulate downstream gene expression, including the release of matrix-degrading enzymes. Although chondrocytes can produce different ROS, the specific mechanisms by which each ROS disrupts cartilage homeostasis are still unclear. Age is now recognized as a major risk factor for OA development, as evidence suggests that ROS produced by Nox2 and Nox4 are involved in chondrocyte differentiation *in vitro* and that ROS produced by chondrocytes are also influenced by aging (29).

The study conducted by Christophe Glorieux and colleagues demonstrated that superoxide (O^2−^) and hydrogen peroxide (H_2_O_2_) are the most common types of ROS (30). O2-disrupts cartilage homeostasis through the effects of age-related factors and excessive mechanical load stimulation, accompanied by a decrease in SOD2, which may exacerbate the typical redox imbalance and ultimately lead to the development of OA (31). Other studies have indeed demonstrated that IL-1β affects NOX4/p22 (phox) expression and ROS production and that NOX4 inhibitors can reduce IL-1β-induced collagenase synthesis in chondrocytes (32). O^2−^ and H_2_O_2_ can be increased by Nox4 stimulation. Nox4 is activated by proinflammatory factors and is considered to be the main active isomer in OA cartilage (33). Moreover, increasing Nox4 may increase ROS levels, exacerbating cartilage destruction. Prevention of OA by detecting the normal threshold of Nox4 is expected to provide a new direction for the treatment of OA.

Oxidative stress is also considered to be an important factor in OA. The expression of three superoxide dismutase (SOD) antioxidant enzymes in OA was investigated by Jenny L Scott et al. (34). Based on the oxidative stress-induced aging model of articular chondrocytes, aged chondrocytes and synovial cells exhibit mitochondrial dysfunction and reduced antioxidant capacity due to decreased activity of the hydrogen peroxide enzymes and SOD and diminished function of peroxidases.

### 3.2 Transcription Factors

Sox9 is a key factor in maintaining cartilage balance, and targeting Sox9 may lead to new OA treatments. Sox9 (35), a transcription factor in the Sox family, stands out due to its high mobility group (HMG)-box DNA binding domain (36). Research on Sox9 and early-stage stem (ES) cells has revealed its role in ligament development. Studies on mouse chimeras have shown that Sox9 is crucial for chondrocyte differentiation and cartilage formation, as cells without Sox9 fail to express important cartilage genes.

Sox9 plays a crucial role in cartilage homeostasis and OA, influencing the development of articular cartilage (37). Research by Oh et al. using ChIP-Seq and RNA sequencing has shown that Sox9 controls chondrocyte ECM cell differentiation by regulating specific ECM genes, such as Acan and Col2a1 (38). Additionally, Ouyang et al. reported that OA patients have lower SOX9 gene expression and higher TNF-α concentrations, while IL-1β decreases SOX9 expression and collagen II levels (39). However, upregulating Sox9 has been shown to alleviate OA symptoms in mice and inhibit inflammatory responses in human chondrocytes. These findings suggested that targeting Sox9 could have therapeutic effects on OA.

Second, Sox9 also influences cartilage homeostasis and OA and can play an important role as a major ECM protein in the growth plate and articular cartilage. To investigate the regulatory role of Sox9 in Acan, Oh et al. used chromatin immunoprecipitation sequencing (ChIP-Seq) and RNA sequencing to screen for the identification of genes affected by Sox9. These results suggest that Sox9 controls chondrocyte ECM cell differentiation by regulating a specific set of ECM genes, including Acan and Col2a1, *in vivo* (11). This affects the development of all articular cartilage.

### 3.3 Growth Factors

Growth factors are known to regulate the activity of chondrocytes; among them, the transforming growth factor beta (TGF-β) family plays an important role in maintaining cartilage homeostasis. Bone morphogenetic proteins (BMPs) and related “growth and differentiation factors” (GDFs) are members of the TGF family (40). These proteins can transmit signals via type I and type II serine–threonine kinase receptors and their intracellular downstream effectors, such as SMAD proteins (40). In the superfamily of TGFs, the availability of a limited number of ligands and receptors leads to a complex interplay of ligand–receptor interactions. This characteristic sets TGFs apart from other growth factor families, contributing to a greater degree of confounding in these interactions. The scarcity of ligands and receptors in the TGF superfamily poses challenges in unraveling the specific binding affinities and functional consequences of these interactions (41). Activation of TGF-βs involves intracellular signaling cascades involving mitogen-activated protein (MAP) kinase p38, ERK-1 and JNK, through which cartilage-specific gene expression is promoted (42).

#### 3.3.1 Transforming growth factor beta 1

Transforming growth factor beta 1 (TGF-β1) plays a role in cartilage and ECM synthesis as a potent inducer and stimulator. TGF-β1 can stimulate the chondrogenic activity of p38, ERK-1 and, to a lesser extent, JNK to initiate and maintain the chondrogenic activity of mesenchymal progenitor cells (MPCs), which may play a role in the treatment of OA (42). In TGF-induced chondrogenesis, the mechanisms of MEK/ERK and p38 kinase β are not yet completely understood (43). Matrix metalloproteinases (MMPs) are known to be involved in the degradation of collagen and aggregated glycans in OA, and tissue inhibitor of metalloproteinase-3 (TIMP-3) is located in the ECM and is the main inhibitor of these enzymes (44). Qureshi, Hamid Yaqoob et al. also demonstrated that Sp1 transcription factors and activation of the ERK–MAPK pathway are crucial for TGF-induced TIMP-3 induction in chondrocytes (45). However, ERK1/2 is an inhibitory component of the TGFβ signaling pathway induced by TGFβ during the process of chondrogenesis (43). Osteoblast-like cells have potential for multiple types of differentiation. Chondrogenesis occurs when these cells are maintained in high-density sedimentation media supplemented with TGF-β1. Activator protein-2 (AP-2) may play a role in chondrogenesis as part of the TGF-β1 and p38 signaling machinery. Treatment of these TGF-β1-stimulated cells with the p38 MAP kinase inhibitor SB203580 rescued AP-2 DNA binding. AP-2 is a target of both the TGF-β1 and p38 MAP kinase signaling pathways, suggesting that there may be a signaling cascade involved (46).

Because the intracellular effector SMAD protein of TGF-β signaling is activated by the corresponding receptor and transferred to the nucleus to regulate transcription, other signaling pathways can also regulate SMAD activation and function at this step; thus, the TGF-β receptor not only regulates SMAD signaling but also allows SMAD-nondependent TGF-β responses (47). TGF-β type I receptors can be activated to stimulate the phosphorylation of SMAD2 and SMAD3, which may subsequently lead to the formation of a heterodimeric complex of SMAD4. TGF-type I receptors may interact with transcriptional coactivators (P/CAF) after SMAD3 activation, as P/CAF can activate SMAD-mediated transcriptional responses independently or in conjunction with p300/CBP (48). However, whether TGF-β/SMAD signaling can be modified through the regulation of P/CAF is unclear. It is also unknown whether TGFβ family members can signal through phosphatidylinositol 3-kinase (PI3K) or the GTPase RhoA, how this signaling occurs and to what extent (47).

#### 3.3.2 Bone morphogenetic protein

Bone morphogenetic protein (BMP) has long been identified as a very comprehensive key regulator of chondrocyte biology (49). BMP proteins are currently considered to constitute the largest subset of the transforming growth factor beta ligand family (50). The osteoconductive activity of TGF-β family members is exclusive to BMPs (51). The MAPK pathway and the SMAD1/5/8 transcription factor pathway are the two downstream pathways through which the BMP receptor complex communicates. The MAPK pathway is activated by direct phosphorylation of type I BMP receptor kinase, but the mechanism of chondrocyte activation is unknown (52). One of the complex features of BMP is the role of the pro-domain in the regulation of osteogenic activity. The common high-affinity docking sites for the BMP-2, −4, −7 and − 10 prepeptides with GDF-5 are the N-terminus of pro-fibronectin-1, and the bone morphogenetic protein/growth and differentiation factor (BMP/GDF) binding site is located within the N-terminal structural domain of pro-fibronectin-1; additionally, rotational shadow electron microscopy of BMP-1 complex molecules bound to pro-fibronectin-7 confirmed the tissue-specific targeting of BMP-4 (53). BMP is a member of the TGF family, but unlike TGFs, it can bind to type I receptors even when there are no type II receptors present. On the other hand, when both type I and type II receptors are present, their binding affinity dramatically increases (54). BMP2 and BMP3 were found to antagonize each other through the TGF-β/activator pathway and by acting as an inhibitor of osteogenic BMP, which suggested that the antagonistic effect may be the result of competition between signaling components common to the TGF-β/activator and BMP pathways (55). As the most abundant BMP in adult bone, BMP3 is a negative determinant of bone mineral density (BMD) (56). BMP2 is required for osteogenic repair and plays a key role in activating skeletal progenitor cells in the periosteum (57).

Ids are inhibitory helix–loop–helix (HLH) transcription factors, and the expression of Id genes in osteoblasts is under the control of calciotropic agents such as BMP and vitamin D. By investigating the role of Id1 and Id3 in osteoblasts in the regulation of bone metabolism *in vivo*, Id1/Id3 heterozygous knockout mice were found to inhibit BMP-induced bone formation *in vivo*, which indicates that Id1 and Id3 are key effectors of BMP-induced osteoblastogenesis (58). However, the complete set of genes regulated by ID proteins during osteoblastogenesis has not been fully identified. SMAD1, SMAD5, and SMAD8 are receptor-regulated SMADs (R-SMADs) that are phosphorylated by activated BMP type I receptors. BMP-6 induces SMAD1 and SMAD5 phosphorylation and nuclear accumulation but not SMAD8 phosphorylation or nuclear accumulation (59). It may function as a transducer of BMP signaling and/or a novel transcriptional regulator (40). The administration of BMP in the treatment of nonunion fractures is thought to accelerate healing and reduce the rate of infection (60).

The large gene regulatory network downstream of the BMP pathway is still relatively underexplored, and its resolution has remained relatively low. A comprehensive understanding of the BMP pathway requires elucidation of the hierarchy of effectors, transcription factors, and regulators and their interactions with other related signaling pathways (50). Next, we identified the signaling pathway that can significantly treat OA.

#### 3.3.3 TGF-β-activated kinase 1

**Figure 1:**
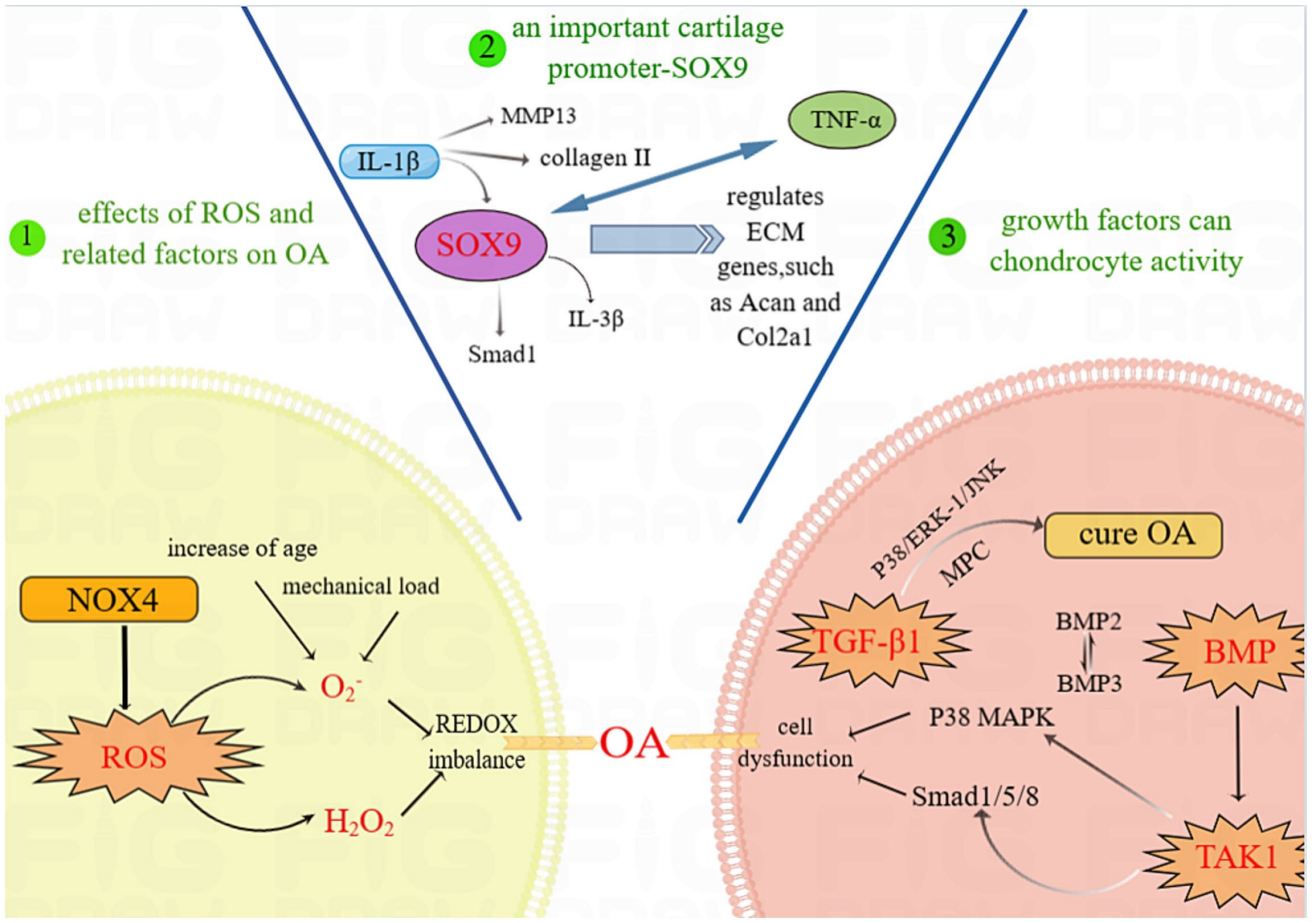
ROS, transcription factors and growth factors involved in cartilage homeostasis. Abbreviations: OA, OA; SOX9, SRY-Box Transcription Factor 9; ROS, Reactive Oxygen Species; NOX4, NADPH oxidase 4; IL-1β, Interleukin-1β; IL-3β, Interleukin-3β; Smad, Mothers against decapentaplegic homolog; MMP13, Matrix Metallopeptidase 13; TNF-α, Tumor Necrosis Factor-α; ECM, cell-extracellular matrix; COL2a1, Recombinant Collagen Type II; TGF-β1, Transforming Growth Factor-β1; p38 MAPK, p38 Mitogen-activated Protein Kinase; BMP, Bone Morphogenetic Protein; JNK, Jun N-terminal kinase; TAK1, TGF-β-activated kinase 1.

TGF-β-activated kinase 1 (TAK1) is a mitogen-activated protein 3 (MAP 3) kinase. TAK1 was demonstrated to be essential for receptor activator of nuclear factor kappa-B ligand (RANKL)-induced osteoclastogenesis by culturing macrophage colony-stimulating factor (M-CSF)-derived monocytes generated from the bone marrow of TAK1-deficient mice in bone marrow lines (61). Deletion of TAK1 appears to affect not only the activation of the p38 MAPK signaling cascade but also the activation of BMP in response to SMAD1/5/8. Further studies have shown that TAK1 can interact with BMP receptors (SMAD1 for S5/S8) at the same site as SMAD465/468/1 and phosphorylate them (52, 62). TAK1 plays a novel role in the development and maintenance of cartilage *in vivo*, and it is also a key mediator of crosstalk between the MAPK and SMAD arms of the BMP signaling pathway (52). These findings also laterally confirm that TAK1 is an important component of the BMP signaling pathway in cartilage. TAK1 serves as a medium for detecting BMP signals. According to Matthew B. Greenblatt et al., TAK1 promotes the phosphorylation of the same site on the BMP receptor, which amplifies SMAD signaling downstream of BMP stimulation, identifying an unidentified crosstalk point between MAPK and SMAD signaling (52). An important regulator of osteoclast formation is receptor activator of nuclear factor-κB (NF-κB) ligand (RANKL), but TAK1 is closely associated with RANKL-induced osteoclast formation (63). A number of potential approaches targeting TAK1 for the treatment of osteolytic diseases have also emerged, and OA is likely to cause osteolytic bone destruction. A TAK1 inhibitor could be used to prevent the spread of OA (Figure 1).

## 4 LncRNA in cartilage protective and destructive

### 4.1 Mitogen-activated protein kinase pathway

**Figure 2:**
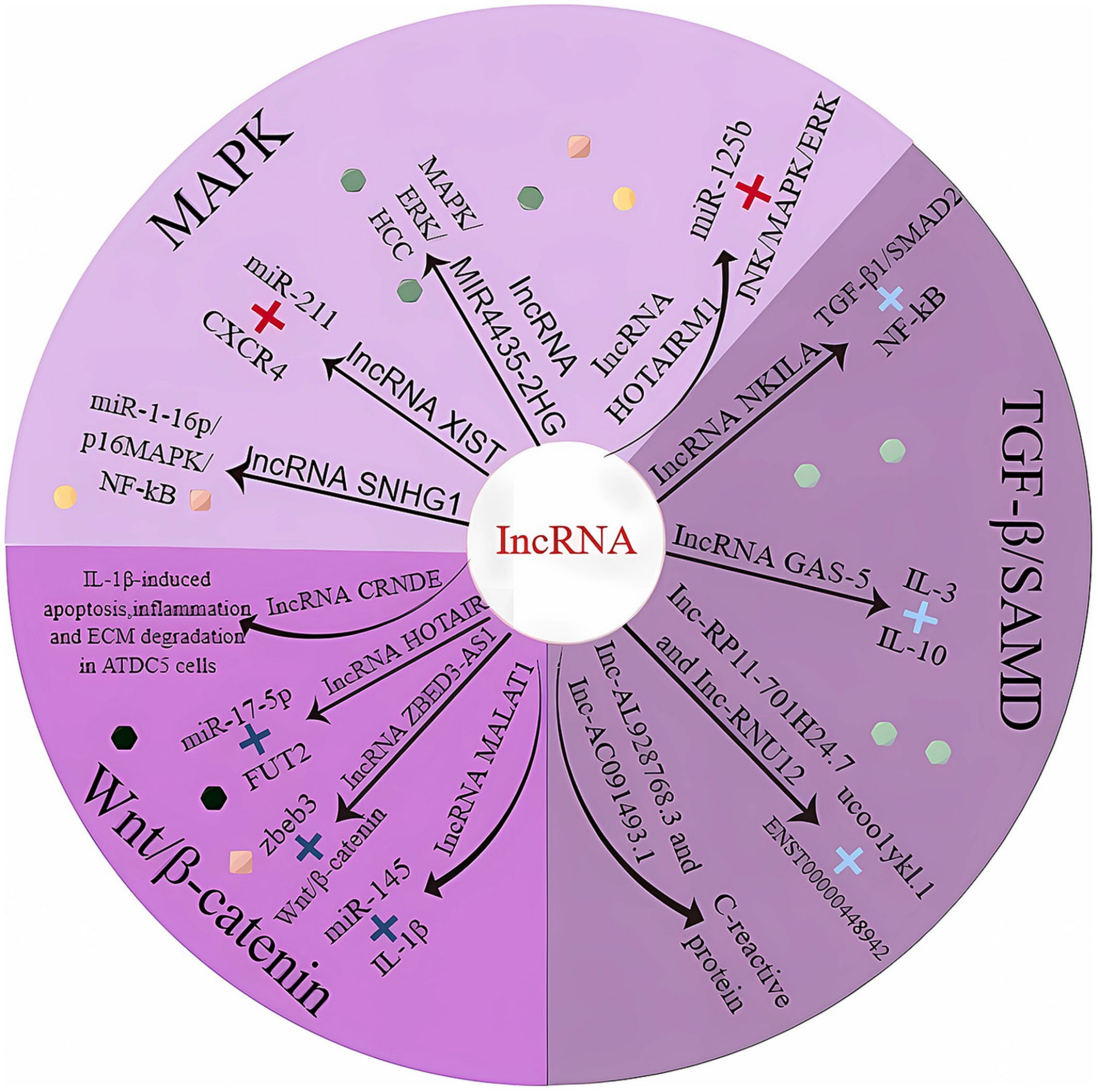
LncRNAs involved in cartilage homeostasis. MAPK, mitogen-activated protein kinase; TGF-β1, transforming growth factor-β1; Smad, mothers against decapentaplegic homolog; CXCR4, motif chemokine receptor 4; NF-Kb, nuclear factor-k-gene binding; ERK, extracellular regulatory protein kinase; HCC, hepatocellular carcinoma; JNK, c-Jun NH2-terminal kinase; IL-3, interleukin 3; IL-10, interleukin 10; IL-1β, interleukin 1β; FUT2, fucosyltransferase 2.

The serine/threonine protein kinase mitogen-activated protein kinase (MAPK) is prevalent in eukaryotic cells, and a dysregulated MAPK signaling pathway accelerates the inflammatory response, which in turn causes the release of large quantities of enzymes that degrade the cartilage matrix and accelerate cartilage degeneration, which in turn causes OA (64). The MAPK subfamily mainly includes p38MAPK, extracellular regulatory protein kinases (ERKs) and c-Jun N-terminal kinases (JNKs). We analyzed the ROS/MAPK signaling pathway as the focus pathway and focused on the p38MAPK signaling pathway in the MAPK pathway (Figure 2) (65).

LncRNA-SNHG1 expression was downregulated in osteogenic inducer-stimulated BMSCs. When overexpressed, LmcRNA-SNHG1 enhanced the interaction of Nedd4 with p-p38, destabilized p-p38, and promoted the ubiquitination of p-p38. The lncRNA SNHG1 negatively regulates the regulatory mechanism of the p38 MAPK signaling pathway through Nedd4-mediated ubiquitination, thereby inhibiting the osteogenic differentiation of BMSCs; these findings were validated in a mouse model of osteoporosis (Table 1) (66). Bioinformatics analysis indicated that miR-211 expression was significantly downregulated and that CXCR4 mRNA expression was upregulated in OA tissues. miR-211 was negatively correlated with XIST and CXCR4, while lncRNA-XIST and CXCR4 were positively correlated in tissue samples. The results suggest that lncRNA-XIST acts as a ceRNA for miR-211 to counteract miR-211-mediated inhibition of CXCR4, thereby regulating chondrocyte proliferation and apoptosis through downstream MAPK signaling (76). *In vitro* experiments with human mesenchymal stem cells (MSCs) revealed that lncRNA-HOTAIRM1-1 was downregulated in OA cartilage and that its downregulation may inhibit the viability of MSCs by regulating the miR-125b/BMPR2 axis, inducing apoptosis and inhibiting differentiation. The JNK/MAPAK/ERK pathway may be a possible downstream mechanism mediating the role of lncRNA-HOTAIRM1-1 in OA development (67).

**Table 1:** LncRNA regulates cartilage homeostasis in OA.

LncRNA	Expression	Function	Model	Reference
*LncRNA SNHG1*	*LncRNA SNHG1*↑ *MiR-1-16p*/p16MAPK/NF-κB↓	pro-inflammatory cytokines in chondrocytes↓ MMPs, ADAMTs, collagen, and aggrecan↓ inflammation of IL-1β-induced OA↓	normal human articular chondrocytes-knee cells	(66)
*LncRNA HOTAIRM1*	*LncRNA HOTAIRM1*↑ *MiR-125b*↓ JNK/MAPK/ERK↓	MSCs viability↓ induce apoptosis and suppress differentiation via	normal articular cartilages	(67)
*LncRNA GAS-5*	*LncRNA GAS-5*↓	IL-13, IL-10↓	peripheral blood mononuclear cells	(68–70)
*LncRNA-NKILA*	L-Glutamine↑ TGF-β1/SMAD2↑ NF-κB↓	TGF-β1 binds to NKILA Phosphorylation of SMAD2/3↑ activation of NF-κB↓	Rat OA chondrocytes	(71)
*LncRNA HOTAIR*	*LncRNA HOTAIR*↑ *MiR-17-5p*↓ FUT2↑	FUT2↑ ECM degradation and chondrocytes apoptosis↑	OA cartilage tissues	(72)
*LncRNA CRNDE*	*LncRNA CRNDE*↓ Il-1β-induced apoptosis, inflammation and ECM degradation in ATDC5 cells↓	*LncRNA CRNDE*↑ Cartilage damage and synovitis in OA rats↓	OA rat model	(73)
*LncRNA MALAT1*	*LncRNA MALAT1*↑*MiR-145*↓ IL-1β↓	*LncRNA MALAT1*↑ *MiR-1*↓ Inhibits IL-1β-induced chondrocyte viability and promotes degradation of cartilage ECM.	cartilage tissue from OA patients and normal cartilage tissue	(74)
*LncRNA ZBED3-AS1*	*LncRNA ZBED3-AS1*↑ ZBED3↑ Wnt/β-catenin↑	chondrification↑	human SFMSCs	(75)

p16 MAPK, p16 mitogen-activated protein kinase; NF-Kb, nuclear factor-k-gene binding; MMP, matrix metallopeptidase; ADAMTs, ADAM metallopeptidase with thrombospondin type 1 motif; IL-1β, interleukin-1β; OA, OA; MAPK, mitogen-activated protein kinase; ERK, extracellular regulatory protein kinase; HCC, hepatocellular carcinoma; CXCR4, motif chemokine receptor 4; JNK, c-Jun NH2-terminal kinase; BMPR2, recombinant bone mitogen-3 receptor 2; IL-10, interleukin-10; IL-13, interleukin-13; TGF-β1, transforming growth factor-β1; NKILA, NF-kappa B-interacting lncRNA; Smad2, mothers against decapentaplegic homolog 2; ECM, cell-extracellular matrix; FUT2, Fucosyltransferase 2; IL-1β, interleukin-1β.

### 4.2 Small mothers against the decapentaplegic pathway

With regard to SMAD, the most studied are the various regulatory effects of TGF-β/SMAD signaling, such as its regulation of adipocyte mesenchymal stem cells (77), its ability to prevent tissue fibrosis (78), its ability to reduce abnormal skin scar formation (79) and its ability to affect SMAD-dependent pathways in infarcted and failing hearts (80), among others. Danielle Kamato et al. performed a signaling pathway-related study of phosphorylation in the SMAD junction region (81). Smad-linked region phosphorylation signaling is initiated by multiple receptors, including G protein-coupled receptors (GPCRs), which act not only as regulators of TGF-β signaling but also as autonomous signaling pathways, indicating that the role of SMAD signaling in cell biology is gradually expanding and implicitly surpassing that of TGF-β signaling (82).

Bioinformatics studies revealed that NKILA expression was reduced in human osteoarthritic cartilage tissues. In addition, dual luciferase assays confirmed that reduced NKILA could act as a ceRNA to improve miR-145 expression, inhibit SP1 expression and regulate the NF-κB signaling pathway, thereby promoting tissue inflammation and inhibiting chondrocyte proliferation and apoptosis (83). Moreover, intragastric administration of L-glutamine to OA model rats has been demonstrated to regulate lncRNA-NKILA expression through the TGF-β1/SMAD2/3 signaling pathway, thereby alleviating OA. Moreover, the experimental conclusions were strengthened by the results obtained in patients with early OA (71).

### 4.3 Wnt/β-catenin pathway

The Wnt/β-catenin signaling pathway is an evolutionarily conserved signaling cascade that has commanding roles in development and adult stem cell maintenance and controls a variety of developmental processes in skeletal and joint patterns; moreover, we hypothesize that the Wnt/β-catenin signaling pathway may be involved in the progression of OA (84, 85). Molecular signatures unique to the Wnt/β-catenin pathway may provide additional insights for therapeutic interventions in arthritis. β-catenin can bind to TCF/LEF transcription factors to activate Wnt target genes in the nucleus, but how extracellular Wnt signaling is transferred to target cells is still unknown; however, it is plausible that Wnt proteins are incorporated into secretory vesicles or exosomes (86–88). Sclerostin and DKK4 are antagonists that inhibit Wnt signaling (89, 90). In one article, the authors asked whether individual Wnts have unique or overlapping functions, and the conclusion that can be drawn from the data available thus far is that the morphological phenotype should correspond to the location of Wnt expression (84). The Wnt/β-catenin signaling cascade engages in crosstalk with RTK/SRK and GPCR-cAMP-PKA signaling cascades to regulate β-catenin phosphorylation and β-catenin-dependent transcription (91). Among the Wnt-related genes, Wnt7b is most closely associated with OA progression according to the currently available information (92). Wnt7b enhances bone marrow mesenchymal stem cell (BMS) self-renewal and osteogenic differentiation through the induction of Sox11, and this trait also closely aligns with OA development (93). At present, many of the specific molecular signatures involved in the development and progression of OA and related forms of arthritis have not been identified, suggesting unlimited possibilities for OA therapy.

Multiple lineage differentiation, flow cytometry, and gain-of-function studies demonstrated that the lncRNA ZBED3-AS1 promotes chondrogenesis in human synovial fluid mesenchymal stem cells (SFMSCs). The lncRNA ZBED3-AS1 directly increased ZBED3 expression, whereas the use of the Wnt inhibitor DKK1 reversed the stimulatory effect of ZBED3-AS1 on chondrogenesis (75). Studies based on anterior cruciate ligament transection to establish a rat model of OA have shown that the lncRNA CRNDE targets and regulates the disheveled-binding antagonist of beta-catenin 1 (DACT1). By recruiting p300, the lncRNA CRNDE promoted the enrichment of H3K27ac in the DACT1 promoter, thereby facilitating DACT1 transcription. In addition, CRNDE impeded the activation of the Wnt/β-catenin pathway in IL-1β-stimulated cells by inducing DACT1 expression (73).

### 4.4 Other pathways

In addition, several lncRNAs can affect OA through other pathways.

The *lncRNA* maternally expressed gene 3 (MEG3) can improve OA by sponging *miR-9-5p*, thereby promoting the expression of the downstream target transcription factor Krüppel-like factor 4 (KLF4), which inhibits apoptosis and inflammatory responses (94).

Extracellular vesicles are small vesicles released by cells that contain proteins, RNA and other biomolecules. These vesicles can influence the function and phenotype of surrounding cells by transporting signaling molecules and genetic material. The extracellular vesicles of bone marrow stem cells contain key signaling molecules and ncRNAs that regulate cartilage homeostasis and can influence chondrocyte activity and function (95).

*L*ong intergenic nonprotein coding *RNA 341* (*LINC00341*) is abnormally downregulated in chondrocytes from OA patient tissues. Qining Yang et al. suggested that this might be one of the factors that contributes to the pathological destruction of cartilage and the increased apoptosis of chondrocytes (96). Further investigation revealed that *LINC00341* interacts with *MiR-141* to stifle its utilitarian restriction to the 3′-untranslated region of YY1-related factor 2 (YAF2) via RNA, and YAF2 is known to be an enemy of apoptotic factors. These authors speculated that the abnormal downregulation of *LINC00341* might lead to YAF2 protein inhibition, which could serve as a windfall for the treatment of OA (96).

## 5 LncRNA-related therapy

### 5.1 Medication

Nonsteroidal anti-inflammatory drugs (NSAIDs) are the first-line therapeutic agents for OA (97), but there are no clinical studies on the impact of NSAIDs on the mechanism of action related to lncRNAs. Zhang and his colleagues found that *lncRNA H19*, combined with nifenazone and agrin (AGR-H19), competitively inhibits TRIM63, thereby improving the treatment of muscular dystrophies for cardiac function (98). It has been reported that *lncRNA H19* is an important *lncRNA* in the pathogenesis of OA. One of these studies showed that knockdown of lncRNA H19 alleviated apoptosis and inflammation by sponging miR130a in LPS-stimulated human C28/I2 chondrocytes (99, 100), and studying the combination of these drugs with nonsteroidal anti-inflammatory drugs is highly important for targeted treatment.

Glucocorticoid receptor activity (GR) is a nuclear receptor that regulates gene transcription and cellular function within cells. Joint cavity injection of glucocorticoids is an effective therapy for OA (101), but the lncRNA-related mechanisms of glucocorticoid therapy for OA have not been studied. GAS5, which is involved in GR activity, is a *lncRNA* of great interest to researchers in recent years (102). GAS5 is an important *lncRNA* in OA patients (99). GAS5 is downregulated in OA patients mainly in articular cartilage tissues and synovial cells. This downregulation may be related to the pathogenesis of OA, and the downregulation of GAS5 may lead to increased cell proliferation, decreased apoptosis, and heightened inflammatory responses, thereby promoting the development and progression of OA (68–70). It has been reported that XIST enhances vascular cell adhesion protein 1 (VCAM-1)-dependent adhesion of monocytes to OA synovial fibroblasts (OASFs), thereby accelerating OA progression (20, 76, 103). Su and her team discovered for the first time that XIST is transcriptionally regulated by GC/GR signaling (104). It will be important to investigate the cellular and molecular mechanisms underlying the effects of glucocorticoids on OA progression via XIST.

Intra-articular injections of hyaluronic acid in OA patients have been reported to be as effective as corticosteroid injections over a 26-week period (105). However, the molecular biological mechanisms associated with lncRNAs have not been studied. Hyaluronic acid (HA) is a ubiquitous extracellular interstitial component that plays a key role in regulating the behavior of cells, including chondrocytes (106). Hyaluronic acid accumulation has been associated with inflammation (107) and cancer (108). The *lncRNA HAS2-AS1* is known to facilitate HAS2 gene expression and HA synthesis, thereby promoting malignant progression in a variety of tumors (109–111). It will be important to investigate the mechanism by which lncRNAs affect OA progression by influencing the metabolism and synthesis of HA.

In addition to clinical first-line drugs, potential drugs that affect OA development through lncRNAs are still being explored.

Brusatol is a drug that inhibits Nrf2-mediated glucose metabolism (112). Gu’s team found that brusatol could partially abrogate the inhibitory effect of *MiR-1323* on *IncRNA ZFAS1* to promote chondrocyte proliferation and inhibit oxidative stress (113). This finding suggested that this compound may be a candidate drug for OA patients with reduced levels of *IncRNA ZFAS1* expression.

Docosahexaenoic acid (DHA) has been shown to have anti-inflammatory and chondroprotective effects on OA chondrocytes (114) and improve chondrogenesis in IL-1β-injured BMSCs via the Wnt/β-catenin and NF-κB pathways. Feng and colleagues reported that DHA, which has dual signaling inhibition properties, may exert its anti-inflammatory, chondroprotective, and chondrogenic effects by regulating *lncRNA MALAT1* levels (74), suggesting that it may be a candidate for OA patients with elevated *lncRNA MALAT1* expression.

L-glutamine (L-Gln) is the amino acid with the highest content in human blood and has anti-inflammatory and antiapoptotic effects (115). High expression of lncRNA-NKILA could alleviate the progression of OA by decreasing the expression of nitric oxide synthase, cyclooxygenase-2, and matrix metalloproteinase-13 (MMP-13), and in a rat OA model, intragastric administration of L-glutamine could mediate the GF-β1/SMAD2/3 signaling pathway and thereby regulate lncRNA-NKILA (71).

*Achyranthes bidentata* polysaccharide (ABPS) is an active component of traditional Chinese medicine, and research has confirmed that ABPSs inhibit chondrocyte apoptosis (116). Fu and his colleagues demonstrated through *in vitro* and *in vivo* experiments that ABPS regulates the expression of the *lncRNAs NEAT1* and *MiR-377-3p* to inhibit ERS in chondrocytes. Likewise, both *the lncRNA NEAT1-related gene set and the miR-377-3p* restraint can weaken the restorative impact of ABPS on trauma centers (117).

By inhibiting the expression of the *lncRNA HOTAIR*, decreasing the protein levels of p-PI3K and p-AKT, and increasing the protein levels of PTEN, APN, and ADIPOR1, Chen’s group discovered that baicalin plays a therapeutic role (72).

Methylene blue (MB) is a synthetic drug that chronically inhibits peripheral nerve axons, thereby reducing or permanently eliminating pain (118). Zheng and colleagues discovered that methylene blue inhibits OA chondrocyte degradation by modulating the expression of the TIMP-1, MMP-1, and MMP-13 proteins (119). Similarly, Li′s team of researchers found that the levels of interleukin 6, tumor necrosis factor-α, interleukin 1β and interleukin 8 were significantly decreased after MB treatment, and MB was used to treat OA-associated pain by upregulating *lncRNA MEG3* levels (120), indicating that OA patients with decreased MEG3 expression might benefit from this treatment.

Xiao’s group discovered that kaempferol regulates the XIST/*MiR-130a*/STAT3 axis in chondrocytes, which in turn inhibits inflammation and extracellular matrix degradation (121). The above studies indicate that brusatol, DHA, L-Gln, ABPS, baicalin, methylene blue and kaempferol are potential therapeutic drugs for OA.

### 5.2 Targeted therapy

There is growing evidence that lncRNAs play a crucial role in OA development (122), and it is worth exploring whether targeting lncRNAs could be a new strategy for the prevention and/or treatment of OA. We refer to published cytological studies and animal studies to identify potential therapeutic targets, as shown in Table 2. We have proposed a strategy for *lncRNA* delivery and targeting based on published animal studies due to the lack of relevant clinical studies.

**Table 2:** Potential lncRNA-related therapy.

Target	Adjustment	Cell Type	Proposed molecular mechanisms	Correlation	Reference
*LncRNA NEAT1*	Downregulate	—	Interaction with miR-377-3p	Inhibits apoptosis of OA chondrocytes	(117)
*LncRNA NEAT1*	Upregulate	MSC	Regulated Sesn2/Nrf2 by interacting with miR-122-5p	Induces the proliferation and autophagy of chondrocytes, inhibits apoptosis	(95)
*LncRNA XIST*	Downregulate	—	*MiR-211*/CXCR4	Inhibits IL-1β-treated chondrocytes cell multiplication and promotes apoptosis	(76)
*LncRNA XIST*	Downregulate	C28/I2 cells	Regulated STAT3 by interacting with *MiR-130a*	Inhibits inflammation and ECM degradation	(121)
*CILinc02*	Downregulate	OA primary cells	Increases the expression of MMP-1 and MMP-13 proteins	Inhibits IL-1, IL-6, IL-17-induced inflammatory responses	(119)
*LncRNA HOTAIR*	Downregulate	—	PTEN/PI3K/AKT pathways	Inhibits OA inflammation	(72)
*LncRNA ZFAS1*	Upregulate	OA primary cells	Interaction with *MiR-1323*	Promotes chondroid proliferation and inhibits oxidative stress	(113)
*LncRNA PILA*	Downregulate	OA primary cells	Regulated TAK1 by methylation	Inhibits chondrocytes apoptosis and ECM breakdown	(123)
*KLF3-AS1*	Upregulate	Human MSCs	Regulated PI3K/Akt/mTOR pathway by Interaction with YBX1	Represses autophagy and apoptosis of IL-1β-treated chondrocytes	(124)
*MM2P*	Upregulate	RAW264.7 cells	Regulated Col1a2 and Acan	Promoted chondrocyte differentiation and function	(125)
*LncRNA H19*	Downregulate	Primary FLSs	Regulated TIMP2 by interacting with miR-106-5p	Promotes chondrocytes proliferation and migration and inhibits ECM degradation	(91)
*LncRNA GAS5*	Upregulate	OA primary cells	Regulated PVT1	ECM degradation and apoptosis	(68–70)
*LncRNA HAS2-AS1*	Downregulate	—	Regulated SIRT1	Inhibition of HA accumulation	(109–111)
*LncRNA MALAT1*	Downregulate	OA primary cells	Regulated Wnt/β-catenin/NF-κB pathway	Represses autophagy and apoptosis of IL-1β-treated chondrocytes	(106)
*LncRNA NKILA*	Upregulate	OA primary cells	Regulated growth factor-β1/SMAD2/3 pathway	Reduced cartilage tissue degradation and MMP-13 expression	(71)
*LncRNA MEG3*	Upregulate	—	Regulated P2X3	Inhibits IL-1β, IL-6, IL-8 and TNF-α induced inflammatory responses	(120)

NEAT1, Nuclear Enriched Abundant Transcript 1; OA, OA; MSC, Mesenchymal Stem Cell; CXCR4, Motif Chemokine Receptor 4; ECM, cell-extracellular matrix; MMP-1, Matrix Metallopeptidase 1; MMP-13, Matrix Metallopeptidase 13; PTEN, Phosphatase and tensin homolog; PI3K, Phosphatidylinositol 3 kinase; AKT, Protein Kinase B; TAK1, TGF-β-activated kinase 1; YBX1, Recombinant Y-Box Binding Protein 1; IL-1β, Interleukin-1β.

Cellular exosomes are expected to be carriers for the selective delivery of target genes to target tissues (126). Cellular/extracellular carriers in blood and synovial fluid are major important pathways for the transport of lncRNAs to the extracellular environment (127–129). Jin and colleagues reported that the exosomal *lncRNA MEG-3* reduced IL-1β-induced chondrocyte senescence and apoptosis to maintain the chondrocyte phenotype (130). The delivery of Sox9 mRNA and protein via exosomes helps chondrocytes differentiate and function (125). In cellular assays, MSCKLF3-AS1-Exos promoted GIT1 expression through phagocytosis of *MiR-206*, thereby mediating the attenuation of chondrocyte damage (131). Cellular experiments suggest that BMSC-derived exosomes may reduce OA-related inflammation by regulating the LYRM4-AS1/GRPR/*MiR-6515-5p* signaling pathway (132). In animal studies, intra-articular injection of exosomes overexpressing *lncRNA H19* was found to promote chondrocyte migration, matrix secretion, apoptosis inhibition and senescence inhibition (133). Wen and colleagues identified a potential component for OA treatment, MSC-Exo-mediated inhibition of the PI3K/Akt/mTOR signaling pathway by KLF3-AS1 through a focus on YBX1 to restrain autophagy and apoptosis in IL-1β-treated chondro-cytes (124). Zhang et al. reported that NEAT1 delivered by BMSC-EVs could delay OA progression by regulating the *MiR-122-5p*/Sesn2/Nrf2 axis *in vivo* (95). The lncRNA SNHG1 (small nuclear RNA host gene 1) inhibits IL-1β in OA by sponging miR-16-5p, thereby inhibiting the downstream p38 MAPK and NF-κB signaling pathways and ultimately IL-1β in OA (134).

Additionally, due to their good biocompatibility and degradability, the use of nanoparticles as effective carriers for targeting lncRNAs offers a novel therapeutic strategy (135). Recent advances in lipid nanoparticles, polymeric nanocarriers and metal-based delivery systems offer new avenues for the delivery of nucleic acid- and lncRNA-based therapeutics (136). Therapeutic vectors, exosomes, nanomaterials, and lentiviruses have great potential as vectors for loading OA therapeutic gene editing systems, although nanoparticle delivery strategies targeting lncRNAs in OA have not been studied experimentally.

Upregulation of lncRNAs appears to be the most common abnormal change reported in the pathogenesis of OA (99). It is reasonable to propose methods to inhibit the expression or activity of these genes. Methods to regulate lncRNA expression *in vivo*, such as locked nucleic acid (LNA) GapmeRs, conventional *siRNAs* and *ASOs*, have been shown to be effective at inhibiting cancer progression (137) and have yet to be tested in animal models of OA. For lncRNA knockdown, it is common knowledge that gene editing enzyme systems such as zinc finger nucleases (ZFNs) and clustered regularly interspaced short palindromic repeats (CRISPR) are significantly superior to RNAi technology (138–140). A few small particle inhibitors have been found to methodically target lncRNA articulation by veiling restricting destinations or vieing for restricting locales or disturbing RNA structure (141). Most of the downstream targets of lncRNA inhibition are proteins (142). It is feasible to propose methods to inhibit the expression or activity of this gene. Protac technology ubiquitinates proteins and has been shown to be effective at inhibiting cancer progression (143, 144). However, these findings need to be tested in animal models of OA, as these findings could lead to the development of new strategies for small-molecule drug therapy for OA.

## 6 Conclusion

As LncRNA research has intensified, a wide variety of evidence suggests that lncRNAs exert different actions through different components of various joints, thus influencing the pathological changes and disease progression of OA. In this paper, we mainly discuss cartilage homeostasis in terms of ROS, transcription factors and growth factors; systematically describe the lncRNA action network related to the MAPK, TGF-β/SMAD, Wnt/β-catenin and other pathways; and summarize the potential therapeutic targets and prospects for involvement in clinical treatment.

LncRNAs are essentially concentrated in OA cartilage tissue and chondrocytes, and it was found that lncRNAs function as miRNA wipes to manage target quality and participate in the guidelines for cartilage digestion and chondrocyte capability. However, this role and the posttranscriptional regulation of target genes/proteins may not explain the full function of lncRNAs in OA. Moreover, various techniques for past investigations of LncRNA articulation and functional parameters, such as the wellspring of test cells, scoring measures, and trial models, may lead to conflicting experimental outcomes. The applicable biomolecular components of different lncRNAs in OA have not been methodically and thoroughly explained.

In addition, the following questions remain unanswered: (1) The reasons for the abnormal regulation of lncRNAs during the occurrence and development of OA are unclear. Inflammation, hypoxia (16) and mechanical stress (145) are the main upstream factors leading to abnormal expression of lncRNAs, but the main factors involved in downstream feedback regulation are unclear. (2) Many *miRNAs* or proteins have been identified as downstream targets of lncRNAs, but their role in OA pathogenesis associated with LncRNA dysfunction has not been determined. (3) Given the diverse biological functions of lncRNAs, it is uncertain whether the role of lncRNAs in the development of OA is tissue specific and/or cell specific. (4) LncRNAs are not accurate enough as biomarkers, and additional lncRNA models containing lncRNAs need to be proposed; however, the corresponding detection methods need to be improved, and other statistical methods, such as predictive values, likelihood ratios, and odds ratios, need to be used according to the purpose of the biomarker under study. Multicenter studies with larger sample sizes need to be conducted to eliminate differences such as ethnicity and sampling bias. (5) The relationships and impacts of lncRNA-related treatments for other diseases need to be studied in greater depth. (6) The effects of combination therapy with multiple lncRNA-related targets need further experimental study.

Although therapeutic nucleic acids have been reported for the treatment of OA, some technical issues, including the underlying mechanisms and effective and specific delivery methods, are not fully understood and have not been developed for OA treatment. Furthermore, the clinical use of lncRNA-based treatments requires more thorough and dependable examination, especially with respect to somewhere safe and secure issues, including immunogenicity, cytotoxicity, and long-term wellbeing (146). Furthermore, the specificity of targeted lncRNAs is very important and needs to be further investigated to avoid off-target side effects. Finally, appropriate targeting of lncRNAs will lead to more effective therapeutic approaches for OA.

## Author contributions

KY: Conceptualization, Supervision, Writing – review & editing. HZ: Writing – original draft. QX: Writing – original draft. HL: Writing – original draft. ZL: Writing – original draft. JD: Writing – review & editing. GG: Conceptualization, Funding acquisition, Supervision, Writing – review & editing.
